# Supplementary material for: Hybrid Models and Biological Model Reduction with PyDSTool
Source: PLoS Comput Biol. 2012 Aug 9;8(8):e1002628. doi: 10.1371/journal.pcbi.1002628 (PMC3415397; doi:10.1371/journal.pcbi.1002628)
Supplement: Text S4 — Complete source code for the PyDSTool package (version 0.88.120504). Includes API documentation and help files linking to web pages. This file is identical to the current public release on Sourceforge.net. (ZIP) [file pcbi.1002628.s004.zip › PyDSTool/html/PyDSTool.Toolbox.optimizers.helpers.quadratic-pysrc.html]

xml version="1.0" encoding="ascii"?


PyDSTool.Toolbox.optimizers.helpers.quadratic


| Home | Trees | Indices | Help | PyDSTool |
| --- | --- | --- | --- | --- |

Package PyDSTool ::
Package Toolbox ::
Package optimizers ::
Package helpers ::
Module quadratic

[hide private]

[frames] | no frames]

# Source Code for Module PyDSTool.Toolbox.optimizers.helpers.quadratic

```
 1   
 2  # Matthieu Brucher 
 3  # Last Change : 2007-07-20 15:04 
 4   
 5  import numpy.linalg 
 6   


7 -class Quadratic(object):


8    """ 
 9    Defines a cost function with a quadratic cost 
10    """ 


11 -  def __init__(self, x, y, f):


12      """ 
13      Creates the function : 
14      - x is the parameters for the function 
15      - y is the data to approximate 
16      - f is an object function defining the gradient and the Hessian if needed 
17        It takes two parameters when called, the values x where the function should be computed and an array params that contains the parameters. The gradient of the function is the gradient for the parameters 
18      """ 
19      self.x = x 
20      self.y = y 
21      self.f = f

22   


23 -  def __call__(self, params):


24      """ 
25      Computes the cost for the specified parameters 
26      """ 
27      return numpy.sum((self.y-self.f(self.x, params))**2)

28   


29 -  def gradient(self, params):


30      """ 
31      Computes the gradient of the function 
32      """ 
33      inter = -2 * self.f.gradient(self.x, params) * (self.y-self.f(self.x, params))[..., numpy.newaxis] 
34      inter.shape=(-1, inter.shape[-1]) 
35      return numpy.sum(inter, axis = 0)

36   


37 -  def hessian(self, params):


38      """ 
39      Compute sthe hessian of the fit function 
40      """ 
41      inter = -2 * self.f.hessian(self.x, params) * (self.y-self.f(self.x, params))[..., numpy.newaxis, numpy.newaxis] + 2 * self.f.gradient(self.x, params)[..., numpy.newaxis] * self.f.gradient(self.x, params)[..., numpy.newaxis, :] 
42      shape = inter.shape[-2], inter.shape[-1] 
43      inter.shape = (-1, inter.shape[-2] * inter.shape[-1]) 
44      temp = numpy.sum(inter, axis = 0) 
45      temp.shape = shape 
46      return temp

47
```

  


| Home | Trees | Indices | Help | PyDSTool |
| --- | --- | --- | --- | --- |

|  |  |
| --- | --- |
| Generated by Epydoc 3.0.1 on Fri May 4 15:24:19 2012 | http://epydoc.sourceforge.net |
